# Effects of pointing movements on visuospatial working memory in a joint-action condition: Evidence from eye movements

**DOI:** 10.3758/s13421-021-01230-w

**Published:** 2021-09-03

**Authors:** Divya Bhatia, Vaishnavi Mohite, Pietro Spataro, Clelia Rossi-Arnaud, Ramesh Kumar Mishra

**Affiliations:** 1 O.P. Jindal Global University, Jindal Institute of Behavioural Sciences, Sonipat, India; 2 University of Hyderabad, Center for Neural and Cognitive Sciences, Hyderabad, India; 3 Department of Economy, Mercatorum University, Rome, Italy; 4 Department of Psychology, Sapienza University, Via dei Marsi 78, 00185 Rome, Italy

**Keywords:** Visuospatial working memory, Eye movements, Pointing movements, Joint action

## Abstract

Previous studies showed that (a) performing pointing movements towards to-be-remembered locations enhanced their later recognition, and (b) in a joint-action condition, experimenter-performed pointing movements benefited memory to the same extent as self-performed movements. The present study replicated these findings and additionally recorded participants’ fixations towards studied arrays. Each trial involved the presentation of two consecutive spatial arrays, where each item occupied a different spatial location. The item locations of one array were encoded by mere visual observation (the no-move array), whereas the locations of the other array were encoded by observation plus pointing movements (the move array). Critically, in Experiment 1, participants took turns with the experimenter in pointing towards the move arrays (joint-action condition), while in Experiment 2 pointing was performed only by the experimenter (passive condition). The results showed that the locations of move arrays were recognized better than the locations of no-move arrays in Experiment 1, but not in Experiment 2. The pattern of eye-fixations was in line with behavioral findings, indicating that in Experiment 1, fixations to the locations of move arrays were higher in number and longer in duration than fixations to the locations of no-move arrays, irrespective of the agent who performed the movements. In contrast, no differences emerged in Experiment 2. We propose that, in the joint-action condition, self- and other-performed pointing movements are coded at the same representational level and their functional equivalency is reflected in a similar pattern of eye-fixations.

## Introduction

In the working memory model originally proposed by Baddeley and Hitch (1974), visuospatial working memory (VSWM) represents the subsystem specifically devoted to the elaboration and maintenance of visual and spatial information (see Baddeley, 2012, for a review). Logie (1995) has further divided this sub-system into a passive visual cache and a movement-based inner scribe associated with rehearsal processes. A large body of research has investigated how movements influence encoding in VSWM and most studies using the dual-task paradigm have reported significant detrimental effects of movement-based secondary tasks when the performed movements were unrelated to the to-be-remembered stimuli (e.g., Baddeley & Lieberman, 1980; Quinn & Ralston, 1986; Rossi-Arnaud, Pieroni, Spataro, & Baddeley, 2012a; Vandierendonck, Kemps, Fastame, & Szmalec, 2004; see Quinn, 2008, for a review). Other studies have, however, demonstrated that pointing movements performed towards the to-be-remembered locations can result in a beneficial effect on visuospatial memory. In particular, in a study by Chum, Bekkering, Dodd, and Pratt (2007), participants were presented with two spatial arrays of circles and squares, where each item was presented at a different location on the screen. The item locations of one array were encoded through mere visual observation (the *no-move array*), while the item locations of the other array were encoded through visual observation accompanied by pointing movements (the *move array*). The results of an immediate memory task showed that the locations of the move arrays were recognized significantly better than no-move arrays – a result later replicated by Dodd and Shumborski (2009, Exp. 1). Chum et al. (2007) explained these findings by proposing that pointing movements promoted a spatial-based perceptual framework, which in turn improved the encoding of the spatial arrangement of to-be-remembered arrays.

Recently, Bhatia et al. (2019b) used the paradigm introduced by Chum et al. (2007) to determine whether observing the pointing movements performed by the experimenter produced the same positive memory consequences as self-performed movements. Collectively, the results of this set of experiments indicated that the pointing movements performed by the experimenter were as beneficial to immediate recognition as self-performed movements when the two types of actions were alternated in a joint-action condition. More specifically, Bhatia et al. (2019b) found that, when participants simply observed the pointing movements performed by the experimenter in a *passive* condition (Experiment 2), the locations of move arrays were recognized no better than no-move arrays. On the other hand, when participants alternated with the experimenter in performing pointing movements in a *joint-action* condition (Experiment 3), then the locations of the arrays pointed by both the participants and the experimenter were recognized better than the locations of the no-move arrays. Bhatia and colleagues (2019b) proposed that, in the joint setting, the arrays pointed by the experimenter were represented in the same functional way as self-pointed arrays, because participants experienced that condition as a social interaction and, therefore, co-represented their partner’s movements as if they were their own.

The results reported by Bhatia et al. (2019b) are intriguing in that they echo previous research demonstrating that working in a joint-action condition leads participants to form shared motor representations that specify the actions that the co-actor is expected to perform (Sebanz, Bekkering, & Knoblich, 2006a; Sebanz, Knoblich, & Prinz, 2005). In line with these studies, Bhatia et al. (2019b) hypothesized that their findings resulted from the co-representation of the actions performed by the experimenter. Specifically, the authors proposed that, in the joint-action condition, the presentation of other-relevant move arrays (i.e., the arrays to which the experimenter had to point) triggered the anticipatory motor simulation of the experimenter's pointing movements (Kourtis, Sebanz, & Knoblich, 2013). They further speculated that action co-representation and anticipatory motor simulation did not occur in the passive observation condition because the experimental setting could not be regarded as a meaningful social interaction. This conclusion was based on previous EEG evidence showing that the way in which social situations are perceived modulates action simulation. Kourtis, Sebanz, and Knoblich (2010), in particular, showed that anticipatory motor activation (as reflected in a larger amplitude of the contingent negative variation) occurred when participants expected a particular action to be performed by a partner with which they were actively interacting, but not when they expected the same action to be performed by a third person with whom they were not interacting. In the Bhatia et al. (2019b)’s study, participants working in the passive condition were seated next to each other but were explicitly instructed to avoid any interaction with the experimenter. Thus, no anticipatory motor activation for the experimenter-pointed arrays could be expected in this condition.

The aim of the present study was to replicate the results reported by Bhatia et al. (2019b) and, most crucially, to provide additional evidence about the cognitive mechanisms underlying the memory advantage associated with experimenter-pointed arrays, by examining the patterns of eye fixations in the joint-action (Experiment 1) and passive-observation (Experiment 2) conditions. The analysis of eye movements has now become a powerful method to understand the nature of the information that is being processed on a moment-to-moment basis (Henderson & Hollingworth, 2003; Hollingworth, Williams, & Henderson, 2001; Zelinsky, Loschky, & Dickinson, 2011; see Ryan & Shen, 2020, for a review). Decades of research have indeed shown that memory can be predicted from the patterns of fixations during the study phase, as the eyes tend to dwell longer on objects that subsequently form long-lasting memory traces (Bylinskii, Isola, Bainbridge, Torralba, & Oliva, 2015; Damiano & Walther, 2019; Liu, Shen, Olsen, & Ryan, 2017; Meghanathan, van Leeuwen, & Nikolaev, 2015; Schwedes & Wentura, 2016). In a study by Pertzov, Avidan, and Zohary (2009), for example, participants were asked to view an array of eight objects before answering questions about the orientation and location of one randomly chosen target object. Across two experiments, the authors found that response accuracy improved with the number of fixations to the target object, suggesting that the proportion of target fixations during encoding are directly related to memory performance. Taken together, these findings suggest that measures of eye movements, such as the number and duration of fixations, can be used as indicators of the quality of memory representations (Hannula et al., 2010; Olejarczyk, Luke, & Henderson, 2014; Ryan, Hannula & Cohen, 2007; Wynn, Shen, & Ryan, 2019).

Interestingly for the present purposes, previous studies demonstrated the existence of a tight coupling between gaze and hand movements, such that gaze proactively guides the hand towards the objects that must be grasped and subsequently towards the landing sites where the objects must be moved (e.g., Ballard, Hayhoe, Li, & Whitehead, 1992; Hayhoe, Bensinger, & Ballard, 1998; Land & Furneaux, 1997; Land, Mennie, & Rusted, 1999; Land, 2006; Land & Hayhoe, 2001). This coupling is so strong that the eye fixations associated with hand-movement planning and control have been regarded as being part of the overall motor programs underlying many common tasks (Land & Furneaux, 1997). In addition, there is now good evidence suggesting that, during action observation, participants produce a pattern of eye fixations that is very similar to that produced when they perform the same action on their own (Flanagan & Johansson, 2003; Rotman, Troje, Johansson, & Flanagan, 2006). For instance, Flanagan and Johansson (2003) compared the pattern of eye movements during the observation and execution of a block-stacking task. Three wooden blocks with different widths had to be stacked from the widest to the narrowest. In the task execution condition, participants fixated each forthcoming grasp and landing site well before the hand arrived; furthermore, their gaze exited the grasp and landing sites at about the same time as the hand. The central finding was that this pattern of gaze-hand coordination was essentially equivalent to that exhibited in the observation condition. Flanagan and Johansson (2003) proposed that the similarity was due to the fact that the participants who observed others’ actions implemented the same motor programs they used during action execution. This co-representation hypothesis is now supported by an increasing amount of data indicating that observing others’ actions can have similar functional and neural effects as performing the same actions on one’s own (Prinz, 1997). For example, the *motor simulation account* assumes that the observation of others’ actions generates an internal “replica” that approximates the motor experience of performed actions (Decety & Grézes, 2006; Jeannerod, 2001). Similarly, the *direct matching hypothesis* proposes that action understanding relies on a mechanism that maps observed actions onto the motor representations of those actions (Iacoboni et al., 1999; Rizzolatti, Fogassi, & Gallese, 2001). Several findings confirm these views. First, the mirror neuron system in the parietal lobe enables us to understand and imitate observed actions through an internal “embodied” simulation that matches action observation with action execution (Enticott, Johnston, Herring, Hoy, & Fitzgerald, 2008; Iacoboni et al., 2005; Rizzolatti & Craighero, 2004; Rizzolatti, Fadiga, Fogassi, & Gallese, 1999). Second, the motor cortex exhibits corresponding neural activity when participants perform specific actions and when they simply observe the same actions being performed by others (Buccino et al., 2001).

Starting from these assumptions, the present study aimed at understanding the cognitive mechanisms through which the pointing movements performed by self and others, in a joint-action condition, come to benefit visuospatial memory. To this purpose, we replicated and extended the experiments reported by Bhatia et al. (2019b) by analyzing the participants’ fixations during the encoding and maintenance phases. More specifically, Experiment 1 in the present study corresponded to the *joint-action* condition introduced by Bhatia and colleagues (2019b, Exp. 3), in which the participant and the experimenter took turns in performing pointing movements towards the item locations of move arrays. On the other hand, Experiment 2 in the present study corresponded to the *passive observation* condition (Bhatia et al., 2019b, Exp. 2), in which participants passively observed the pointing movements performed by the experimenter. In addition to replicating the behavioral findings obtained by Bhatia et al. (2019b), we predicted that, in the joint-action condition, the memory advantage for the locations of self-pointed arrays should be reflected in the eye-fixation measures. That is, given the functional link between eye movements self-pointed arrays should be fixated more often and for longer durations than item locations in the no-move arrays. Second, considering the strong similarity between the eye movements triggered by performed and observed actions (Flanagan & Johansson, 2003), we expected that, in the joint-action condition, the pattern of eye fixations obtained when participants pointed towards the move arrays should be very similar to that obtained when they observed the pointing movements performed by the experimenter. That is, if the presentation of other-relevant arrays involves the anticipatory motor simulation of the experimenter's movements (Decety & Grézes, 2006; Iacoboni et al., 1999; Jeannerod, 2001; Rizzolatti et al., 2001), then it follows that the locations of the experimenter-pointed arrays should be also fixated more frequently and for longer durations compared to the locations of the no-move arrays. On the other hand, no difference in eye fixations towards the locations of move and no-move arrays should be obtained in the passive observation condition, as participants working in this condition were not expected to simulate the experimenter’s pointing movements (Kourtis et al., 2010).

## Experiment 1

Experiment 1 used the same procedure as in the study by Bhatia et al. (2019b, Exp. 3): during each trial in the encoding phase participants had to remember the locations of two arrays – a no-move array plus a move array. A joint-action condition was adopted, in which participants alternated with the experimenter in performing pointing movements towards the locations of move arrays. Then, in the recognition phase, they had to determine whether a probe array matched either the move or the no-move array presented at encoding. Behaviorally, we expected that the locations of both self-pointed and experimenter-pointed arrays should be recognized better than the locations of no-move arrays (Bhatia et al., 2019b). We also predicted that participants should fixate the locations of self-pointed arrays more than those of no-move arrays. Finally, if participants working in this condition simulated the movements performed by the experimenter (Bhatia et al., 2019b), then a similar pattern of eye fixations should emerge in the trials in which pointing movements were performed by the experimenter – that is, participants should fixate the locations of experimenter-pointed arrays more frequently and for longer durations than the locations of no-move arrays. In other words, we expected that the interaction between Condition (move vs. no-move arrays) and Agent Cue (participant- vs. experimenter-performed pointing movements) should be non-significant.

## Method

### Participants

Twenty naive volunteers (16 males; age: *M* = 23.35 years, *SD* = 2.20 years) from the University of Hyderabad (India) participated in the experiment. All the participants reported having normal or corrected-to-normal vision. The institutional ethics committee of the University of Hyderabad approved the study. In the Bhatia et al. (2019b) study, the effect size associated with the significant main effect of Condition (showing the memory advantage of move arrays) in the joint-action condition of Experiment 3 was *η*_p_^2^ = 0.41, which corresponds to *f* = 0.83. Using the G*Power software (Faul, Erdfelder, Lang, & Buchner, 2007), we estimated that, with *N* = 20, *α* = 0.05 and a medium correlation between the repeated measures (*r* = 0.50), the post hoc power to achieve a within-subjects effect of Condition of a magnitude similar to that obtained by Bhatia et al. (2019b) exceeded 0.99 in a repeated ANOVA (*F* tests: ANOVA repeated measures, within factors).

### Apparatus and stimuli

**Fig. 1:**
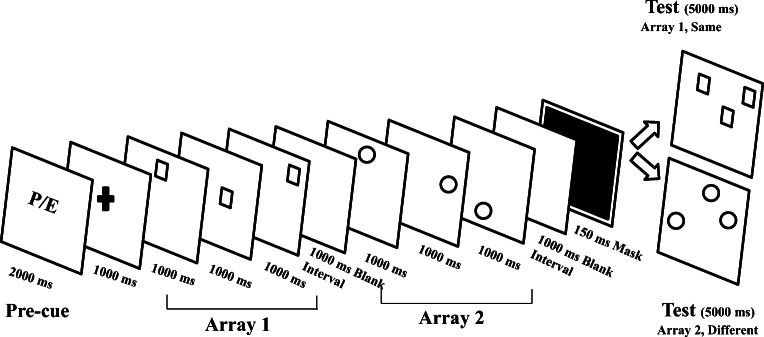
Schematic overview of the procedure used in Experiment 1. During the encoding phase, participants were initially presented with a letter cue indicating who had to perform the pointing movements (P for the participant, E for the experimenter). Then, they studied two consecutive arrays including three (or four) circles or squares: depending on the instructions, one array was designed as the no-move array, while the other array was designed as the move array. Each item was presented for 1000 ms. A mask (a black screen) followed the encoding phase for 150 ms, after which participants saw an array of squares or an array of circles: they had 5000 ms to decide whether the test array matched one of the two arrays presented at encoding

The stimuli were the same as those used by Bhatia et al. (2019b). The whole set comprised 192 visuospatial arrays, containing three or four items, each item appearing at a different location out of a 5 × 5 matrix, which could not be seen by participants. Of these, 96 arrays contained only circles (48 for each array size), whereas the other 96 arrays contained only squares. Both the circles and the squares were 2 cm × 2 cm in size, with two adjacent items separated by 1 cm (see Rossi-Arnaud, Spataro, & Longobardi, 2012b, for examples). For the test phase, we constructed, for each array, a test lure having all the item locations in common with the original array except one, which was shifted by one or two cells. All the stimuli were displayed in black against a grey background, including the fixation cross and the letter cues for the pointing instructions (‘P’ for the participant, ‘E’ for the experimenter; both displayed in Times New Roman, size 40 pt), which indicated who had to perform the pointing movements towards the locations of the upcoming move array (see Fig. 1). Responses were collected using a Cedrus RB-844 response pad (Cedrus Corporation, San Pedro, CA, USA).

The experiment was conducted in a noise-free, dimly lit room. Participants sat at 40 cm from a desktop mounting a SR Research Eyelink 1000 eye tracker (SR Research Ltd, Ontario, Canada), which recorded eye movements with a sampling rate of 1,000 Hz and a spatial resolution less than 0.01°. A chin rest was used to stabilize head movements during the experiment. The experimenter was seated on a raised chair placed either on the right or on the left of the participant (counterbalanced across participants), so that hand movements did not obstruct the camera. A 15-in. HP display monitor (1,280 × 1,024 pixels; refresh rate: 60 Hz), controlled by Experiment Builder software (SR Research Ltd, Ontario, Canada), displayed the stimuli. To locate the positions of the eye fixations, the display was divided into 25 regions of interest, corresponding to the 5 × 5 matrix in which the stimuli were originally displayed. Squares and circles were placed at the center of each cell, so that its boundaries formed the corresponding region of interest for the eye-tracking analysis.

### Design and procedure

Experiment 1 followed a 2 (Array Order: first vs. second) × 2 (Array Size: 3 vs. 4 items) × 2 (Condition: no-move vs. move) × 2 (Agent Cue: P-cued vs. E-cued) within-subjects design. The general procedure was modelled after Bhatia et al. (2019b, Experiment 3), which in turn was modelled after Chum et al. (2007) and Dodd and Shumborski (2009) (see Fig. 1). Each trial comprised two phases: an initial encoding phase, in which two arrays of three or four items were presented (the move array and the no-move array), and a recognition phase in which participants had to determine whether the locations of a probe array matched the locations of one of the two encoded arrays. During the encoding phase, each trial started with a letter cue for 2,000 ms, which signaled who had to point to the items in the move array (‘P’ for the participant, ‘E’ for the experimenter). The letter cue was followed by a fixation cross at the center of the screen for 1,000 ms, after which two spatial arrays were consecutively presented, one after the other. Both arrays contained the same number of items (either three or four) but differed in shape (for instance, if the first array contained circles, then the second array contained squares, and vice versa). Each item in the array was presented for 1,000 ms, such that each subsequent circle (or square) appeared when the previous circle (or square) disappeared. Thus, the entire array was presented for a total of 3,000 ms or 4,000 ms, depending upon the array size. The sampling of the items’ locations within each array was completely random (with the constraints that, within a given trial, no item could occur in a location previously occupied by another item and two successive items could not occupy adjacent cells). A blank screen appeared for 1,000 ms at the end of each array, during which participants could maintain the encoded array in working memory. As mentioned in the *Introduction*, the inclusion of these blank screens was justified by a growing body of research showing that, when people want to keep information active in working memory, they often allocate visual attention to the empty locations in space in which the stimuli were presented during the encoding phase (see Theeuwes, Belopolsky, & Olivers, 2009, for a review). Participants were instructed to passively view the locations of the presented items for the no-move arrays and to move their hands towards the locations of each item until touching the screen for the move array; in both cases, they were instructed to remember the locations of each item in view of a subsequent recognition task. We randomly divided the participants into two groups, such that one group pointed at the circle arrays (and passively viewed the square arrays), whereas the other group pointed at the square arrays (and passively viewed the circle arrays). In half the trials, the no-move array was presented first, followed by the move array; for the other half of the trials, the order was reversed. The participant and the experimenter took turns to perform pointing movements towards the item locations of the move arrays, as indicated by the cue (P/E) presented at the beginning of each trial. The experiment comprised 48 trials for each array size, for a total of 96 trials. Array size was blocked and the order of the blocks was counterbalanced across participants such that half of the participants performed the three-item trials before the four-item trials, while the other half of the participants followed the opposite order. A break of 2 min was given after completion of the first block of trials.

The recognition phase began immediately after the presentation of the second array, starting with a mask for 150 ms. The purpose of the recognition task was to examine the participant’s memory for the locations of the items presented either in the move or in the no-move array. Thus, each test screen contained either squares or circles. Participants did not know in advance which array was tested in each trial: thus, they had to memorize both the arrays presented during the study phase. They were instructed to judge whether the locations of the items in the test array matched (or not) those shown at encoding, by pressing two large keys labeled “same” or “different.” Participants were required to make a response within 5,000 ms.

It should be noted that the experimenter (VM) who performed the pointing movements in the E-cued trials was aware of the results reported by Bhatia et al. (2019b) and did not vary across participants.

### Data analysis

For recognition memory, accuracy was computed as the mean percentage of arrays correctly recognized as being equal to those shown during the encoding phase (this is the same dependent variable used in previous studies: Bhatia et al., 2019b; Chum et al., 2007; Dodd & Shumborski, 2009). Data were analyzed with a series of 2 (Array Order: first vs. second) × 2 (Array Size: 3 vs. 4 items) × 2 (Condition: move vs. no-move) × 2 (Agent Cue: P-cued vs. E-cued) repeated-measures ANOVAs. Considering the complexity of a design with four independent factors and the fact that our interest was specifically focused on the differences between move and no-move arrays (i.e., on the effects of Condition), we have limited our discussion in the following sections to the main effects of all factors and the highest-order interactions involving the Condition factor. A detailed illustration of the results obtained in each analysis is reported in Table 1.

**Table 1:** Experiment 1: Full results of the mixed 2 (Condition: move vs. no-move arrays) × 2 (Agent: P-cued vs. E-cued trials) × 2 (Order: first vs. second array) and 2 (Size: 3-item vs. 4-item arrays) ANOVAs. Significant effects and interactions (p < 0.05) are highlighted in bold

Main effects and interactions	Recognition accuracy		Fixation % encoded arrays		Fixation % blank screen		Gaze durations encoded arrays		Gaze durations blank screen	
	F Test	p level	F Test	p level	F Test	p level	F Test	p level	F Test	p level
CONDITION	*F*=7.97	**0.011**	*F*=16.46	**0.001**	*F*=10.63	**0.004**	*F*=22.88	**0.000**	*F*=13.81	**0.001**
AGENT	*F*=0.24	0.628	*F*=0.46	0.503	*F*=0.01	0.931	*F*=8.33	**0.009**	*F*=0.70	0.411
ORDER	*F*=20.10	**0.000**	*F*=4.22	**0.054**	*F*=5.84	**0.026**	*F*=0.62	0.439	*F*=12.21	**0.002**
SIZE	*F*=0.01	0.929	*F*=11.36	**0.003**	*F*=96.55	**0.000**	*F*=16.20	**0.001**	*F*=60.73	**0.000**
CONDITION*AGENT	*F*=3.94	0.062	*F*=19.38	**0.000**	*F*=0.34	0.562	*F*=5.40	**0.031**	*F*=0.62	0.437
CONDITION*ORDER	*F*=2.01	0.172	*F*=1.19	0.289	*F*=1.95	0.178	*F*=3.15	*0.092*	*F*=3.11	0.094
CONDITION*SIZE	*F*=0.55	0.466	*F*=11.47	**0.003**	*F*=4.84	**0.040**	*F*=7.04	**0.016**	*F*=3.78	0.067
AGENT*ORDER	*F*=0.73	0.403	*F*=1.72	0.205	*F*=8.46	**0.009**	*F*=37.78	**0.000**	*F*=5.72	**0.027**
AGENT*SIZE	*F*=1.05	0.317	*F*=0.01	0.918	*F*=14.96	**0.001**	*F*=23.75	**0.000**	*F*=10.19	**0.005**
ORDER*SIZE	*F*=0.07	0.790	*F*=95.95	**0.000**	*F*=0.47	0.501	*F*=50.23	**0.000**	*F*=1.12	0.303
CONDITION*AGENT*ORDER	*F*=15.74	**0.001**	*F*=21.74	**0.000**	*F*=0.69	0.414	*F*=11.41	**0.003**	*F*=0.66	0.427
CONDITION*AGENT*SIZE	*F*=2.57	0.125	*F*=7.36	**0.014**	*F*=0.00	0.975	*F*=0.39	0.538	*F*=2.77	0.112
CONDITION*ORDER*SIZE	*F*=0.40	0.534	*F*=4.29	**0.052**	*F*=1.52	0.233	*F*=0.06	0.800	*F*=1.00	0.328
AGENT*ORDER*SIZE	*F*=0.16	0.689	*F*=0.04	0.832	*F*=21.41	**0.000**	*F*=21.41	**0.000**	*F*=16.33	**0.001**
CONDITION*AGENT*ORDER*SIZE	*F*=3.53	0.075	*F*=5.31	**0.033**	*F*=1.58	0.223	*F*=1.83	0.192	*F*=1.92	0.182

For eye movements, we computed the *relative fixation percentages* and the *relative gaze durations* for both the encoded arrays and the blank screen. By definition, a fixation represents a period during which the eyes remain relatively still and information is encoded into working memory (specifically, the eyes had to remain fixed on a given position for more than 80 ms; Eyelink 1000 user manual, SR Research Ltd, Ontario, Canada), whereas gaze duration refers to the sum of all the fixations falling within an area of interest (AOI; Rayner, 2009). In the current study, the AOIs were obtained by dividing the display into a 5 × 5 matrix, corresponding to that in which the items were originally presented. Relative fixation percentages were computed by dividing the total number of fixations falling in a given AOI by the total number of fixations to the entire screen and multiplying the result by 100. Likewise, relative gaze duration was computed by summing the durations of all the fixations falling in a given AOI, dividing it by the cumulative duration of the fixations to the entire screen, and multiplying the result by 100 (Chang & Choi, 2014; d’Ydewalle & De Bruycker, 2007; Georgescu et al., 2013).

## Results and discussion

### Recognition accuracy

**Fig. 2:**
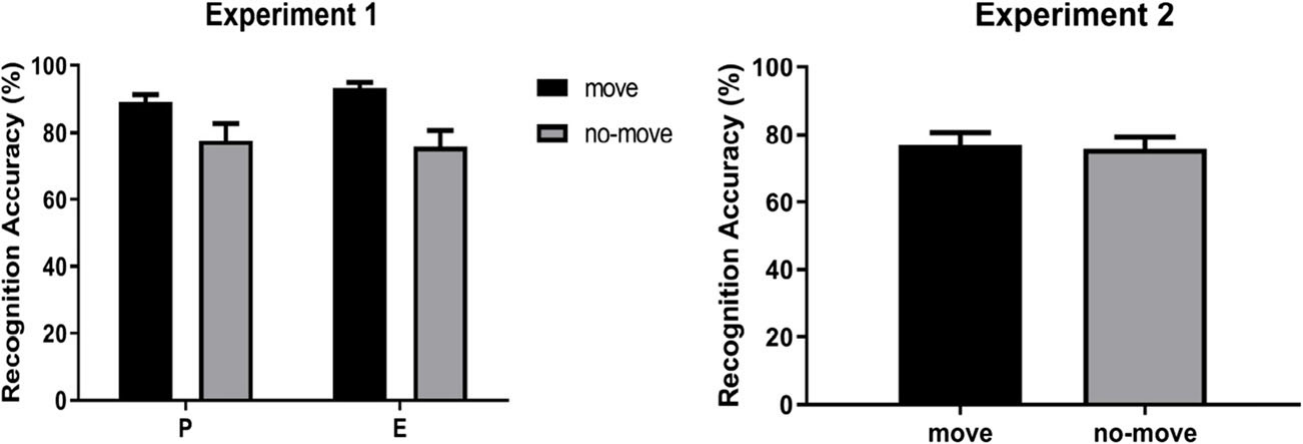
Mean recognition accuracy in Experiments 1 and 2, as a function of Condition (move vs. no- move) and Agent Cue (P-cued vs. E-cued trials). Bars represent standard errors

For recognition accuracy, the full analysis (see Table 1) revealed significant main effects of Condition [*F*(1, 19) *=* 7.97, *MSE* = 518.07, *p* = 0.011, *η*_p_^2^ = 0.29], indicating that recognition accuracy was higher for move (*M* = 91.14%) than for no-move arrays (*M* = 76.76%), and Array Order [*F*(1, 19) *=* 20.10, *MSE* = 541.73, *p* < 0.001, *η*_p_^2^ = 0.51], indicating that recognition accuracy was higher for arrays presented as second (*M* = 89.79%) than for arrays presented as first (*M* = 78.12%). The critical interaction between Condition and Agent Cue did not reach the standard level of significance [*F*(1, 19) *=* 3.94, *MSE* = 172.53, *p* = 0.062, η_p_^2^ = 0.17]. In fact, move arrays were recognized better than no-move arrays in both the P-cued [*M* = 89.16% vs. *M* = 77.70%: *F*(1, 19) *=* 4.42, *p* = 0.049, η_p_^2^ = 0.19] and E-cued trials [*M* = 93.12% vs. *M* = 75.83%: *F*(1, 19) *=* 11.31, *p* = 0.003, η_p_^2^ = 0.37] (see the left panel of Fig. 2). However, the three-way interaction between Condition, Agent Cue, and Order was significant [*F*(1, 19) *=* 15.74, *MSE* = 285.88, *p* = 0.001, η_p_^2^ = 0.45]. A follow-up analysis of simple effects indicated that, for P-cued trials, the advantage of the move condition was significant for arrays presented as second [*M*(move) = 96.67% vs. *M*(no-move) = 79.99%: *F*(1, 19) *=* 8.63, *p* = 0.008, η_p_^2^ = 0.31], but not for arrays presented as first [*M*(move) = 81.66% vs. *M*(no-move) = 75.41%: *F*(1, 19) *=* 0.93, *p* = 0.34, η_p_^2^ = 0.05]. In contrast, for E-cued trials, the advantage of the move condition was significant for arrays presented as first [*M*(move) = 91.24% vs. *M*(no-move) = 64.16%: *F*(1, 19) *=* 25.77, *p* < 0.001, η_p_^2^ = 0.58], but not for arrays presented as second [*M*(move) = 94.99% vs. *M*(no-move) = 87.49%: *F*(1, 19) *=* 1.64, *p* = 0.21, η_p_^2^ = 0.08].

### Relative fixation percentages for the encoded arrays

**Fig. 3:**
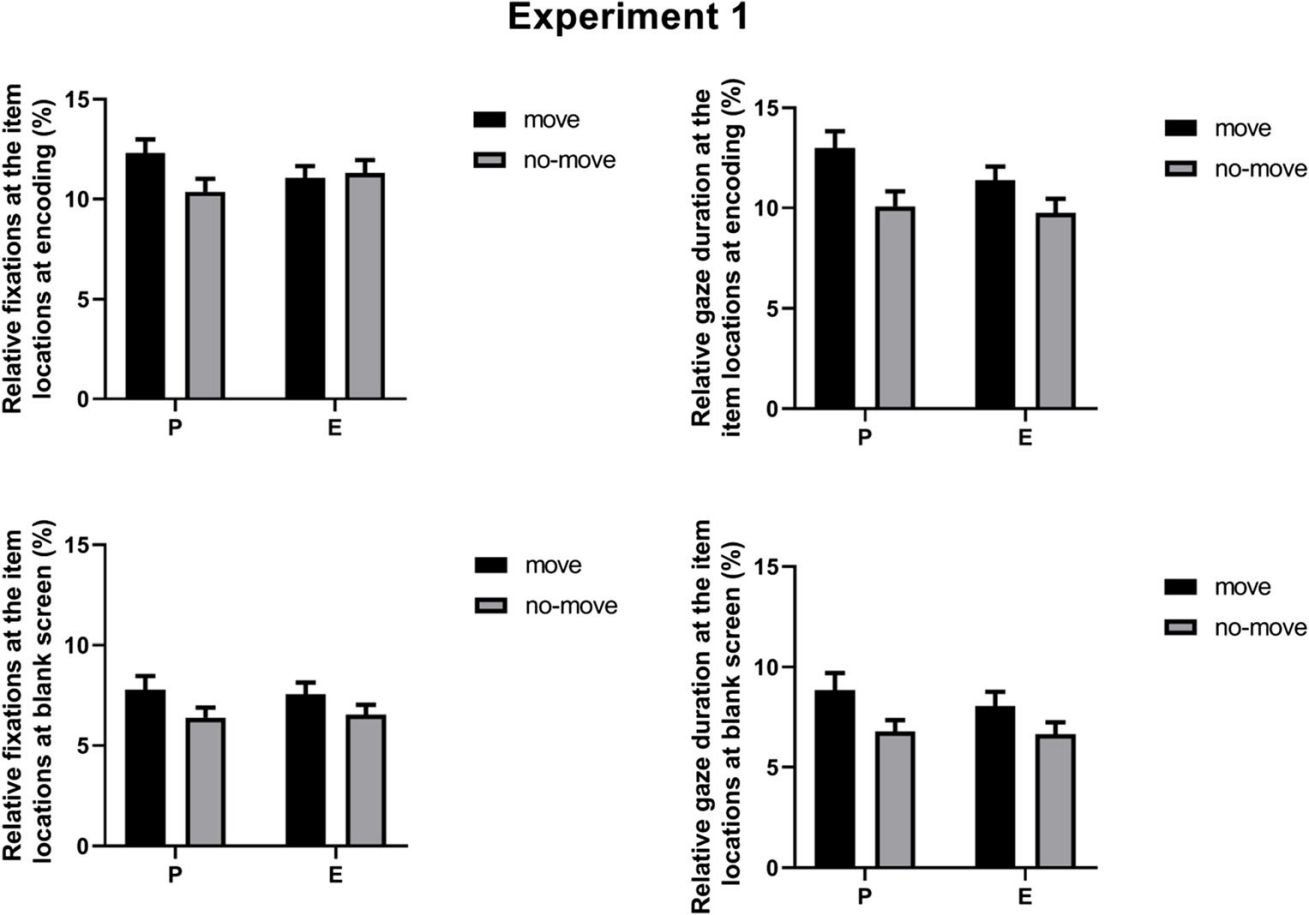
Experiment 1: Mean relative fixation percentages and gaze duration for the encoded arrays (**top panel**) and the blank screen (**bottom panel**), as a function of Condition (move vs. no- move) and Agent Cue (P-cued vs. E-cued trials). Bars represent standard errors

For encoded arrays, the full analysis on relative fixation percentages (see Table 1) revealed: (a) a significant main effect of Condition [*F*(1, 19) *=* 16.46, *MSE* = 0.88, *p* = 0.001, η_p_^2^ = 0.46], indicating that relative fixation percentages were higher for move (*M* = 11.69%) than for no-move arrays (*M* = 10.84%); (b) a significant main effect of Order [*F*(1, 19) *=* 4.22, *MSE* = 5.12, *p* = 0.054, η_p_^2^ = 0.18], indicating that relative fixation percentages were slightly higher for arrays presented as second (*M* = 11.53%) than for arrays presented as first (*M* = 11.01%); (c) a significant main effect of Size [*F*(1, 19) *=* 11.36, *MSE* = 5.52, *p* = 0.003, η_p_^2^ = 0.37], indicating that relative fixation percentages were higher for four-item arrays (*M* = 11.71%) than for three-item arrays (*M* = 10.82%). These effects were qualified by a two-way interaction between Condition and Agent Cue [*F*(1, 19) *=* 19.38, *MSE* = 4.94, *p* < 0.001, η_p_^2^ = 0.51] and a four-way interaction between Condition, Agent Cue, Order, and Size [*F*(1, 19) *=* 5.31, *MSE* = 1.71, *p* = 0.033, η_p_^2^ = 0.22]. For the two-way interaction, a follow-up analysis of simple effects revealed that relative fixation percentages were higher for move than for no-move arrays in the P-cued trials [*M*(move) = 12.32% vs. *M*(no-move) = 10.37%: *F*(1, 19) *=* 23.02, *p* < 0.001, η_p_^2^ = 0.55], but not in the E-cued trials [*M*(move) = 11.07% vs. *M*(no-move) = 11.31%: *F*(1, 19) *=* 1.26, *p* = 0.28, η_p_^2^ = 0.06] (see the upper left panel of Fig. 3). For the four-way interaction, a follow-up analysis of simple effects showed that relative fixation percentages were higher for move than for no-move arrays in all conditions, with the exception of three-item P-cued arrays presented as first [*M*(move) = 9.85% vs. *M*(no-move) = 8.89%: *F*(1, 19) *=* 2.91, *p* = 0.10, η_p_^2^ = 0.13] and three-item E-cued arrays presented as first [*M*(move) = 9.66% vs. *M*(no-move) = 9.10%: *F*(1, 19) *=* 1.13, *p* = 0.30, η_p_^2^ = 0.06].

### Relative fixation percentages for the blank screen

For the blank screen, the full analysis on relative fixation percentages (see Table 1) revealed: (a) a significant main effect of Condition [*F*(1, 19) *=* 10.63, *MSE* = 2.73, *p* = 0.004, η_p_^2^ = 0.36], indicating that relative fixation percentages at the blank screen were higher for move (*M* = 7.67%) than for no-move arrays (*M* = 6.47%); (b) a significant main effect of Array Order [*F*(1, 19) *=* 5.84, *MSE* = 11.83, *p* = 0.026, η_p_^2^ = 0.24], indicating that relative fixation percentages at the blank screen were higher for arrays presented as first (*M* = 7.54%) than for arrays presented as second (*M* = 6.61%); (c) a significant main effect of Size [*F*(1, 19) *=* 96.55, *MSE* = 8.01, *p* < 0.001, η_p_^2^ = 0.84], indicating that relative fixation percentages were higher for three-item arrays (*M* = 8.63%) than for four-item arrays (*M* = 5.51%). These effects were qualified by a significant two-way interaction between Condition and Size [*F*(1, 19) *=* 4.84, *MSE* = 4.99, *p* = 0.040, η_p_^2^ = 0.20]. A follow-up analysis of simple effects showed that relative fixation percentages at the blank screen were higher for move than for no-move arrays for three-item arrays [*M*(move) = 9.50% vs. *M*(no-move) = 7.75%: *F*(1, 19) *=* 13.65, *p* = 0.002, η_p_^2^ = 0.42], but not for four-item arrays [*M*(move) = 5.84% vs. *M*(no-move) = 5.19%: *F*(1, 19) *=* 2.49, *p* = 0.13, η_p_^2^ = 0.12]. The critical interaction between Condition and Agent Cue was not significant [*F*(1, 19) *=* 0.34, *p* = 0.56, η_p_^2^ = 0.02], suggesting that relative fixation percentages at the blank screen were higher for move than for no-move arrays in both the P-cued [*M*(move) = 7.79% vs. *M*(no-move) = 6.39%: *F*(1, 19) *=* 6.27, *p* = 0.021, η_p_^2^ = 0.24] and E-cued trials [*M*(move) = 7.56% vs. *M*(no-move) = 6.54%: *F*(1, 19) *=* 6.41, *p* = 0.020, η_p_^2^ = 0.25] (see the bottom left panel of Fig. 3).

### Relative gaze durations for the encoded arrays

For the encoded arrays, the full analysis on relative gaze durations (see Table 1) revealed significant main effects of Condition [*F*(1, 19) *=* 22.88, *MSE* = 18.00, *p* < 0.001, η_p_^2^ = 0.55], Agent Cue [*F*(1, 19) *=* 8.33, *MSE* = 9.09, *p* = 0.009, η_p_^2^ = 0.31], and Array Size [*F*(1, 19) *=* 16.20, *MSE* = 7.29, *p* = 0.001, η_p_^2^ = 0.46]. An inspection of the means indicated that relative gaze durations were higher for move (*M* = 12.18%) than for no-move arrays (*M* = 9.92%), higher in the P-cued (*M* = 11.54%) than in the E-cued trials (*M* = 10.56%), and higher for four-item (*M* = 11.66%) than for three-item arrays (*M* = 10.44%). These effects were qualified by a two-way interaction between Condition and Agent Cue [*F*(1, 19) *=* 5.40, *MSE* = 6.16, *p* = 0.031, η_p_^2^ = 0.22] and a three-way interaction between Condition, Agent Cue and Array Order [*F*(1, 19) *=* 11.41, *MSE* = 1.34, *p* = 0.003, η_p_^2^ = 0.38]: however, in both cases, the follow-up analyses of simple effects indicated that relative gaze durations at encoding were higher for move than for no-move arrays in all conditions [*F*(1, 19) *>* 17.51, *p* ≤ 0.001, η_p_^2^ > 0.48 and *F*(1, 19) *>* 10.35, *p* < 0.005, η_p_^2^ > 0.35, respectively] (see the upper right panel of Fig. 3).

### Relative gaze durations for the blank screen

For the blank screen, the full analysis on relative gaze durations (see Table 1) revealed significant main effects of Condition [*F*(1, 19) *=* 13.81, *MSE* = 17.58, *p* = 0.001, η_p_^2^ = 0.42], Array Size [*F*(1, 19) *=* 60.73, *MSE* = 13.15, *p* < 0.001, η_p_^2^ = 0.76], and Array Order [*F*(1, 19) *=* 12.21, *MSE* = 15.57, *p* = 0.002, η_p_^2^ = 0.39]. An inspection of the means indicated that relative gaze durations were higher for move (*M* = 8.46%) than for no-move arrays (*M* = 6.72%), higher for three-item (*M* = 9.17%) than for four-item arrays (*M* = 6.01%), and higher for arrays presented as first (*M* = 8.36%) than for arrays presented as second (*M* = 6.82%). These effects were qualified by marginal two-way interactions between Condition and Array Size [*F*(1, 19) *=* 3.78, *MSE* = 6.47, *p* = 0.067, η_p_^2^ = 0.17] and between Condition and Array Order [*F*(1, 19) *=* 3.11, *MSE* = 9.74, *p* = 0.094, η_p_^2^ = 0.14]. However, the follow-up analyses of simple effects indicated that relative gaze durations at the blank screen were higher for move than for no-move arrays in all conditions [*F*(1, 19) *>* 4.98, *p* < 0.038, η_p_^2^ > 0.21 and *F*(1, 19) *>* 5.61, *p* < 0.029, η_p_^2^ > 0.23, respectively]. The critical interaction between Condition and Agent Cue was not significant [*F*(1, 19) *=* 0.62, *p* = 0.43, η_p_^2^ = 0.03], suggesting that relative gaze durations at the blank screen were higher for move than for no-move arrays in both the P-cued [*M*(move) = 8.86% vs. *M*(no-move) = 6.78%: *F*(1, 19) *=* 6.90, *p* = 0.017, η_p_^2^ = 0.27] and the E-cued trials [*M*(move) = 8.06% vs. *M*(no-move) = 6.66%: *F*(1, 19) *=* 10.66, *p* = 0.004, η_p_^2^ = 0.36] (see the bottom right panel of Fig. 3).

In summary, the behavioral results of Experiment 1 confirmed the conclusions previously reached by Bhatia et al. (2019b), showing that in a joint-action condition in which participants alternated with the experimenter in making pointing movements, move arrays were recognized better than no-move arrays; furthermore, the advantage occurred for both self- and experimenter-pointed arrays. At the same time, there were two notable exceptions. The first is that the effect of Array Size was not significant in the present study, whereas Bhatia et al. (2019b, Exp.3) reported a memory advantage for three-item arrays (over four-item arrays). This finding challenges the idea that pointing movements affect visuospatial memory in a load-dependent manner (Chum et al., 2007), a conclusion that was already put into question by Dodd and Shumborski (2009). The second exception is that we found a three-way interaction between condition, agent cue, and array order that was not apparent in the study by Bhatia et al. (2019, Exp.3). Given that this interaction was not previously observed and that a numerical advantage for move arrays occurred in all conditions, we are reluctant to provide a theoretical interpretation. Clearly, more research is needed to ascertain the replicability of this specific outcome.

Turning to the analyses of fixation percentages and gaze durations, our predictions were substantially supported, since participants fixated the move arrays more frequently and for longer durations than the no-move arrays in both the P-cued and the E-cued trials (the only exception being the relative fixation percentages for the encoded arrays, which did not differ between the move and no-move arrays in the E-cued trials). Interestingly, we also found that the effects of Array Order and Array Size varied as a function of the type of array being analyzed. Specifically, we found that relative fixation percentages during the encoding phase were higher for arrays presented as second than for arrays presented as first (consistent with the recognition advantage of the former arrays). However, this pattern was reversed when we analyzed the relative fixation percentages at the blank screen. Similarly, both the relative fixation percentages and the relative gaze durations were higher for four-item arrays than for three-item arrays when the analyses were limited to the encoding phase; in contrast, they were higher for three-item than for four-item arrays when the analyses were focused on the blank screen. The way in which these differences contribute to memory performance is actually unclear: however, it seems clear that the functional role of the eye movements performed during the encoding phase might be different from that played by eye movements performed during the appearance of the blank screen (Theeuwes et al., 2009).

## Experiment 2

Experiment 2 used a passive observation condition in which all the pointing movements were performed by the experimenter and passively observed by participants. The results previously reported by Bhatia et al. (2019b) suggest that observing the experimenter’s pointing movements should have no positive influence on the recognition of the locations of move arrays. Since Experiment 2 used the same stimuli and the same procedure illustrated by Bhatia et al. (2019b), we expected to replicate their findings. We also expected no difference in the fixation and gaze duration measures between move and no-move items, suggesting that participants would not simulate the experimenter’s movements in this passive condition.

## Method

### Participants

A new sample of 20 naive volunteers (13 males, mean age: *M* = 24.1 years, *SD* = 3.44) from the University of Hyderabad (India) students’ community participated in the experiment. They all reported having normal or corrected-to-normal vision. The study was conducted with the approval of the institutional ethics committee of the University of Hyderabad.

### Apparatus and stimuli

The apparatus and the stimuli were the same as described in Experiment 1.

### Design and procedure

Experiment 1 followed a 2 (Array Order: first vs. second) × 2 (Array Size: 3 vs. 4 items) × 2 (Condition: no-move vs. move) within-subjects design.

The general procedure mirrored that adopted in Experiment 1, with the exception that, during the encoding phase, participants were instructed to passively observe the pointing movements performed by the experimenter. At the beginning of the experimental session, they were explicitly warned to hold their hands still on the table. Half the participants observed the experimenter making pointing movements towards the squares’ locations, while the other half observed the experimenter making pointing movements towards the circles’ locations. The experimenter who performed the pointing movements (VM) was the same as in Experiment 1 and did not vary across participants.

### Data analysis

Dependent variables were the same as those illustrated in Experiment 1 (percentage accuracy for recognition memory, relative fixation percentages, and relative gaze durations for the encoded arrays and the blank screen). They were analyzed with a series of 2 (Array Order: first vs. second) × 2 (Array Size: 3 vs. 4 items) × 2 (Condition: no-move vs. move) repeated-measures ANOVAs. As in Experiment 1, we have limited our discussion to the main effects of all factors and the highest-order interactions involving the Condition factor. Table 2 reports the full results of each analysis.

**Table 2:** Experiment 2: Full results of the mixed 2 (Condition: move vs. no-move arrays) × 2 (Agent: P-cued vs. E-cued trials) × 2 (Order: first vs. second array) and 2 (Size: 3-item vs. 4-item arrays) ANOVAs. Significant effects and interactions (p *< 0.05) are highlighted in bold*

Main effects and interactions	Recognition accuracy		Fixation % encoded arrays		Fixation % blank screen		Gaze durations encoded arrays		Gaze durations blank screen	
	F Test	p level	F Test	p level	F Test	p level	F Test	p level	F Test	p level
CONDITION	*F*=0.25	0.622	*F*=0.42	0.523	*F*=0.06	0.807	*F*=1.11	0.305	*F*=0.01	0.915
ORDER	*F*=9.50	**0.006**	*F*=3.68	0.070	*F*=18.07	**0.000**	*F*=3.42	0.080	*F*=20.95	**0.000**
SIZE	*F*=8.16	**0.010**	*F*=9.84	**0.005**	*F*=81.84	**0.000**	*F*=13.71	**0.002**	*F*=57.19	**0.000**
CONDITION*ORDER	*F*=0.004	0.948	*F*=0.13	0.713	*F*=0.00	0.990	*F*=0.13	0.721	*F*=0.91	0.352
CONDITION*SIZE	*F*=0.52	0.478	*F*=0.37	0.550	*F*=0.003	0.956	*F*=0.50	0.485	*F*=0.94	0.344
ORDER*SIZE	*F*=4.31	**0.052**	*F*=87.54	**0.000**	*F*=0.01	0.893	*F*=92.82	**0.000**	*F*=0.18	0.671
CONDITION*ORDER*SIZE	*F*=0.75	0.396	*F*=0.20	0.656	*F*=0.43	0.516	*F*=0.37	0.548	*F*=0.26	0.610

## Results and discussion

### Recognition accuracy

The full analysis (Table 2) revealed significant main effects of Array Size [*F*(1, 19) *=* 8.16, *MSE* = 173.88, *p* = 0.01, η_p_^2^ = 0.30] and Array Order [*F*(1, 19) *=* 9.50, *MSE* = 753.08, *p* = 0.006, η_p_^2^ = 0.33], suggesting that recognition accuracy was higher for three-item (*M* = 79.33%) than for four-item arrays (*M* = 73.37%) and higher for arrays presented as second (*M* = 83.04%) than for arrays presented as first (*M* = 69.66%). The main effect of Condition was not significant [*F*(1, 19) *=* 0.25, *MSE* = 201.51, *p* = 0.62, η_p_^2^ = 0.01; see the right panel of Fig. 2], as they were all the interactions involving the Condition factor [*F*(1, 19) < 0.75, *p* < 0.39]. This null result was supported by two types of follow-up analyses. First, we ran a Bayesian analysis by collapsing data across array size and array order, which showed that the null hypothesis was 5.19 times more likely than the alternate hypothesis (Rouder, Speckman, Sun, Morey, & Iverson, 2009). Second, to determine whether we had sufficient power to detect a significant difference, we conducted a post hoc power analysis. The to-be reached effect size was estimated from Experiment 1 by taking into account the means and standard deviations of the move and no-move arrays in the E-cued trials: *d*_z_ = 0.85. Using the software G-Power (Faul et al., 2007), we estimated that, with *N* = 18 and *α* = 0.05, the power to detect an effect of Condition comparable to that observed in Experiment 1 was 0.95 (*t*-test for dependent means, two tails).

### Relative fixation percentages for the encoded arrays

**Fig. 4:**
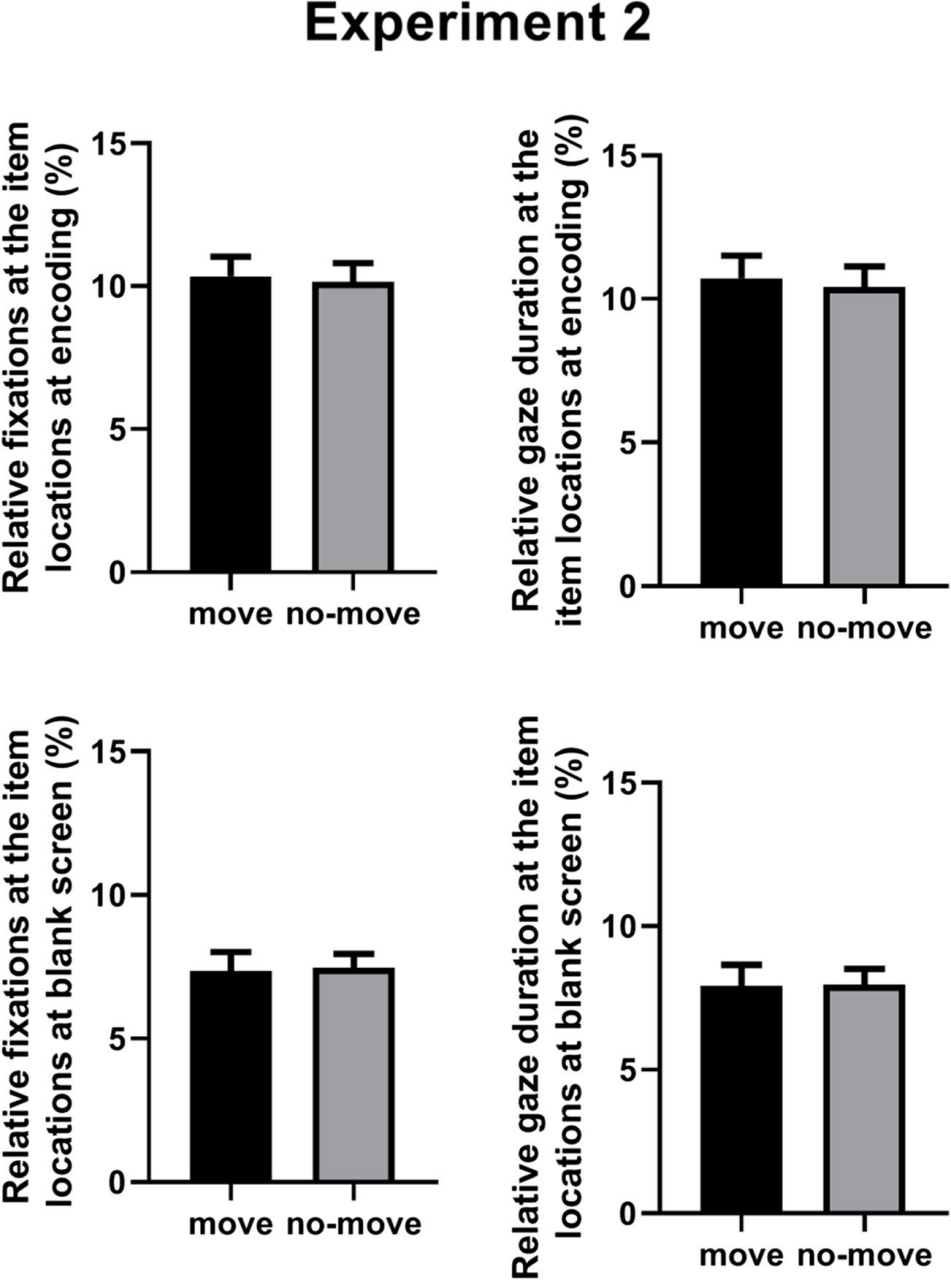
Experiment 2: Mean relative fixation percentages and gaze duration for the encoded arrays (top panel) and the blank screen (bottom panel), as a function of Condition (move vs. no- move). Bars represent standard errors

The full analysis (Table 2) revealed a significant main effect of Array Size [*F*(1, 19) *= 9.84*, *MSE* = 5.32, *p* = 0.005, η_p_^2^ = 0.34]. Relative fixation percentages were higher for four-item (*M* = 10.82%) than for three-item arrays (*M* = 9.68%). The main effect of Condition was not significant [*F*(1, 19) *=* 0.42, *MSE* = 3.03, *p* = 0.52, η_p_^2^ = 0.02; see the left upper panel of Fig. 4], as they were all the interactions involving the Condition factor [*F*(1, 19) *<* 0.37, *p* > 0.55]. The Bayesian paired-sample *t*-test confirmed that the null hypothesis was 4.78 times more likely than the alternate hypothesis.

### Relative fixation percentages for the blank screen

The full analysis (Table 2) revealed significant main effects of Array Size [*F*(1, 19) *=* 81.84, *MSE* = 4.96, *p* < 0.001, η_p_^2^ = 0.81] and Array Order [*F*(1, 19) *= 18.07*, *MSE* = 6.63, *p* < 0.001, η_p_^2^ = 0.48]. Relative fixation percentages were higher for three-item (*M* = 9.01%) than for four-item arrays (*M* = 5.82%) and higher for arrays presented as first (*M* = 8.28%) than for arrays presented as second (*M* = 6.55%). The main effect of Condition was not significant [*F*(1, 19) *=* 0.06, *MSE* = 7.27, *p* = 0.80, η_p_^2^ = 0.003; see the left bottom panel of Fig. 4], as they were all the interactions involving the Condition factor [*F*(1, 19) *<* 0.43, *p* > 0.51]. The Bayesian paired-sample *t*-test confirmed that the null hypothesis was 5.69 times more likely than the alternate hypothesis.

### Relative gaze durations for the encoded arrays

The full analysis (Table 2) revealed a significant main effect of Array Size [*F*(1, 19) *=* 13.71, *MSE* = 6.87, *p* = 0.002, η_p_^2^ = 0.41]. Relative gaze durations were higher for four-item (*M* = 11.33%) than for three-item arrays (*M* = 9.79%). The main effect of Condition was not significant [*F*(1, 19) *=* 1.11, *MSE* = 3.77, *p* = 0.30, η_p_^2^ = 0.05; see the right upper panel of Fig. 4], as they were all the interactions involving the Condition factor [*F*(1, 19) *<* 0.50, *p* > 0.48]. The corresponding Bayesian paired-sample *t*-test indicated that the null hypothesis was 3.48 times more likely than the alternate hypothesis.

### Relative gaze durations for the blank screen

The full analysis (Table 2) revealed significant main effects of Array Size [*F*(1, 19) *=* 57.19, *MSE* = 7.86, *p* < 0.001, η_p_^2^ = 0.75] and Array Order [*F*(1, 19) *=* 20.95, *MSE* = 9.16, *p* < 0.001, η_p_^2^ = 0.52]. Relative gaze durations were higher for three-item (*M* = 9.62%) than for four-item arrays (*M* = 6.27%) and higher for arrays presented as first (*M* = 9.04%) than for arrays presented as second (*M* = 6.85%). The main effect of Condition was not significant [*F*(1, 19) *=* 0.01, *MSE* = 10.16, *p* = 0.91, η_p_^2^ = 0.001; see the right bottom panel of Fig. 4], as they were all the interactions involving the Condition factor [*F*(1, 19) *<* 0.94, *p* > 0.34]. The corresponding Bayesian paired-sample *t*-test indicated that the null hypothesis was 5.83 times more likely than the alternate hypothesis.

### Cross-experiment analysis

To further determine whether recognition performance in the E-cued trials varied as a function of the different conditions of Experiments 1 and 2 (joint condition vs. passive observation), a cross-experimental analysis was performed. For Experiment 1, only the data from E-cued trials were used in this analysis. A mixed 2 (Condition: move vs. no-move array) × 2 (Experiment: Exp. 1 vs. Exp. 2) ANOVA showed that the critical two-way interaction was significant [*F*(1, 38*) =* 8.30*, p =* 0.006, η_p_^2^ = 0.18]. A follow-up analysis of simple effects confirmed that the locations of move arrays were recognized significantly better than the locations of no-move arrays in Experiment 1 [*F*(1, 38) *=* 19.00, *p* < 0.001, η_p_^2^ = 0.33], but not in Experiment 2 [*F*(1, 38) *=* 0.08, *p* = 0.78, η_p_^2^ = 0.002].

Similar ANOVA analyses were performed for the dependent variables reflecting eye movements. For the relative fixation percentages at the encoded arrays, the two-way interaction between Condition and Experiment was not significant [*F*(1, 38*) =* 1.45*, p =* 0.23, η_p_^2^ = 0.037]. However, the same interaction approached the marginal significance level when analyzing the relative fixation percentages at the blank screen [*F*(1, 38*) =* 3.68*, p =* 0.06, η_p_^2^ = 0.09], confirming that participants fixated the locations previously occupied by move arrays more than those occupied by no-move arrays in Experiment 1 [*F*(1, 38) *=* 6.05, *p* = 0.019, η_p_^2^ = 0.137], but not in Experiment 2 [*F*(1, 38) *=* 0.06, *p* = 0.80, η_p_^2^ = 0.002].

Lastly, the analyses performed on the relative gaze durations for the encoded arrays and the blank screen showed that the two-way interactions between Condition and Experiment were again significant [*F*(1, 38*) =* 7.90*, p =* 0.008, η_p_^2^ = 0.17 and *F*(1, 38*) =* 4.83*, p =* 0.034, η_p_^2^ = 0.11, respectively]. The following analyses of simple effects found that, in both cases, relative gaze durations were higher for move than for no-move arrays in Experiment 1 [*F*(1, 38) *=* 24.67, *p* < 0.001, η_p_^2^ = 0.39 and *F*(1, 38) *=* 8.94, *p* = 0.005, η_p_^2^ = 0.19, respectively], but not in Experiment 2 [*F*(1, 38) *=* 0.98, *p* = 0.32, η_p_^2^ = 0.025 and *F*(1, 38) *=* 0.01, *p* = 0.91, η_p_^2^ = 0.00, respectively].

In summary, Experiment 2 replicated the behavioral findings reported by Bhatia et al. (2019b), in that the passive observation of the pointing movements performed by the experimenter did not enhance recognition memory for move arrays (relative to no-move arrays). In agreement, the analysis of eye movements revealed no difference between move and no-move arrays in terms of fixation percentages and relative gaze durations. Taken together, the results obtained in Experiments 1 and 2 support the hypothesis that participants simulated the movements performed by the experimenter in a joint-action condition in which the two agents alternated in performing the pointing movements, but not in a passive observation condition. Such a conclusion was further reinforced by the cross-experimental analysis of participants’ performance in E-cued trials.

With respect to the effects of array size and array order, the present results were fully consistent with the conclusions reached by Bhatia et al. (2019b, Exp.3), showing significant memory advantages for shorter over longer arrays and for second-presented over first-presented arrays (similar findings have been previously reported by Chum et al., 2007, and by Dodd & Shumborski, 2009). In addition, the analyses of relative fixation percentages and relative gaze durations revealed a reversed pattern, similar to that illustrated in Experiment 1. Specifically, both the fixation percentages and gaze durations were higher for four-item than for three-item arrays during the encoding phase; in contrast, during the appearance of the blank screen, the locations previously occupied by three-item arrays were fixated more often and for longer durations, as compared to the locations previously occupied by four-item arrays. This pattern is again consistent with the idea that fixations to the encoded arrays and blank screens have different roles that may be differentially linked to attention and memory processes (Czoschke, Henschke, & Lange, 2019).

## General discussion

The present study investigated the effects of pointing movements on VSWM in a joint-action condition, by simultaneously recording the participants’ fixations during the encoding phase. We employed a paradigm introduced by Chum et al. (2007), in which each trial involved the presentation of two subsequent arrays: a no-move array, encoded through visual observation only, plus a move array, encoded through visual observation accompanied by pointing movements. The task was to determine whether a probe array, presented in a later test phase, matched or not one of the two previously encoded arrays. Behaviorally, the analysis of the recognition performance replicated the pattern reported by Bhatia et al. (2019b). When the participants and the experimenter alternated in performing pointing movements (Experiment 1), move arrays were recognized more accurately than no-move arrays, irrespective of the agent who pointed (i.e., both the participant- and the experimenter-pointed arrays enjoyed a significant advantage). In contrast, when participants passively observed the pointing movements performed by the experimenter (Experiment 2), move arrays were recognized no better than no-move arrays. Most importantly, the memory differences between move and no-move arrays were accurately reflected in the pattern of eye fixations. In the joint-action conditions of Experiment 1, both the fixation percentages and the relative gaze durations were higher for move than for no-move arrays, with the advantage applying to participant- and experimenter-pointed arrays to the same extent. This pattern contrasted with the results obtained in the passive conditions of Experiment 2, in which neither the fixation percentages nor the gaze durations differed between move and no-move arrays.

It has been suggested that visual attention has a critical role in guiding the motor system (Hoffman & Nelson, 1981; Hoffman & Subramaniam, 1995; Posner, 1980). More specifically, several studies used eye fixations as a measure of the amount of attention paid to the stimuli (Godfroid, Boers, & Housen, 2013; Godfroid & Uggen, 2013; Rayner, 2009) and showed that the number and/or the duration of fixations were highly predictive of subsequent memory (Chaffin, Morris, & Seely, 2001; Vainio, Hyönä, & Pajunen, 2009). For instance, in a study by Godfroid et al. (2013), participants were asked to read short paragraphs containing pseudo-words while their eye movements were being recorded. They found that fixations to the pseudo-words were longer than those to matched control words and significantly predicted their recognition in a later test (see Godfroid & Uggen, 2013, for similar findings). Since we found that both fixation percentages and gaze durations were higher for move than for no-move arrays, we can speculate that the move arrays pointed by participants were allocated a higher amount of visual attention, resulting in a better memory representation of these arrays.

However, the most important finding of the present study is that, in the joint-action conditions of Experiment 1, the pattern of fixations in the experimenter-pointing trials was very similar to that observed in the participant-pointing trials: move arrays were associated with higher fixation percentages and longer gaze-durations than no-move arrays, irrespective of the agent who performed the pointing movements. Such a result indicates that the move arrays pointed by the experimenter attracted greater attention than no-move arrays, just like self-pointed arrays. The explanation that we favor is that participants working in the joint-action condition incorporated the partner’s actions in their motor programs, as if they were their own (Bhatia et al., 2019b). Support for this hypothesis comes from a study by Kourtis, Knoblich, Woźniak, and Sebanz (2014), which investigated attention allocation and self/other action representation during the planning phase of a joint-action task (synchronously lifting and clinking glasses). The joint action condition was compared with two other conditions requiring unimanual individual actions or bimanual individual actions. Attention allocation processes were examined by recording two lateralized EEG components, namely the anterior attention negativity and the late attention positivity, whereas action planning processes were investigated by recording the late contingent negative variation and the movement-related potential. The analysis of the first two components showed that the early stages of joint action planning involved dividing attention between the locations relevant to one’s own part of the task and the locations relevant to the partner’s part of the task. That is, participants took into account the locations and the objects that required their partner’s attention, even if they were only relevant to the co-actor. Most interestingly for the present purposes, Kourtis et al. (2014) found that the late contingent negative variation and the movement-related potential were larger when participants were planning an action to participate in a joint task, compared with planning the same action to act alone. The larger amplitudes of these components in the joint-action condition (particularly relevant over the left premotor areas) suggest that, in addition to planning one’s own actions, participants anticipated and represented the co-actor’s actions at an effector-unspecific level.

According to the direct-matching hypothesis, the observation of others’ actions causes an automatic resonance in the observer’s motor system, allowing him (or her) to understand the outcomes of these actions through simulation (Gallese, Keysers, & Rizzolatti, 2004; Rizzolatti, Fogassi, & Gallese, 2001). From this perspective, our results are in line with the hypothesis advanced by Bhatia et al. (2019b) that participants working in the joint-action condition (but not those working in the passive condition) simulated and co-represented the experimenter’s pointing movements as if they were on their own command (Atmaca et al., 2008, 2011; Sebanz et al., 2003, 2006). As mentioned above, Flanagan and Johansson (2003) found that the pattern of eye fixations was highly similar when performing and observing the same task and proposed that participants working in the action-observation conditions implemented motor programs equivalent to those used in the action-execution condition (see also Rotman, Troje, Johansson, & Flanagan, 2006). The present study expands this evidence by showing that, in the joint-action condition, participants fixated the move arrays more often and for longer durations than the no-move arrays both when they performed the pointing movements and when they simply observed the movements performed by the experimenter. Although we did not record the EEG components and thus we cannot provide direct evidence in support of the involvement of primary motor areas in action planning, the similarity of our results with those reported by Flanagan and Johansson (2003) suggests that the direct-matching hypothesis can provide a viable explanation in both cases.

As predicted, the results obtained in the passive conditions of Experiment 2 were very different from those reached in the active condition of Experiment 1. First, the arrays pointed by the experimenter were recognized no better than no-move arrays. Second, the experimenter-pointed arrays did not enjoy a significant advantage in terms of fixation percentages or gaze durations. These results suggest that the way in which the arrays pointed by the experimenter were processed depended on whether the participant was actively involved in the task or not. In agreement, previous electrophysiological studies have shown that the social relationship between the participant and the co-actor plays a crucial role in the ability to anticipate others' actions (Hogeveen & Obhi, 2012). Specifically, anticipatory motor activation, as indexed by the amplitude of the contingent negative variation, was found to be stronger when participants expected a specific action to be performed by an interacting partner than by a third person they did not interact with (Kourtis, Sebanz, & Knoblich, 2010, 2013). These findings indicate that participants were able to simulate the actions performed by the interaction partner, but not those performed by a loner (Tsai, Sebanz, & Knoblich, 2011; Vesper, Butterfill, Knoblich, & Sebanz, 2010). A similar explanation could apply to the passive observation conditions of Experiment 2, in which the absence of any personal involvement from the participant's side might have hindered their ability to perceive the experimenter as an interaction partner. If this were the case, then the fact that the pointing movements performed by the experimenter were not co-represented in the participants’ motor system would come as little surprise.

Our explanation is based on the idea that participants working in the joint-action conditions of Experiment 1 (but not those working in the passive conditions of Experiment 2) covertly simulated and co-represented the pointing movements performed by the experimenter, and this resulted in a similar pattern of eye fixations towards the move and no-move arrays. There are, however, at least two alternative hypotheses that we cannot rule out. One potential account is based on previous evidence showing that visual attention for the space near the hand is prioritized, leading to faster detection of visual targets appearing close to one's own hand (Reed, Grubb, & Steele, 2006; Tseng & Bridgeman, 2011). The study by Sun and Thomas (2013) investigated whether such facilitation also occurred for the information presented near the hands of another actor. Their results demonstrated that the mere presence of another's hand in a passive observation condition was not sufficient to bias attention, since participants were no faster to detect targets that appeared close to their friend's hand than targets appearing away from the friend's hand. However, when the participants and their friend performed a joint action task together, a significant facilitation was observed for targets presented both near one’s own hands and near the friend’s hands. In agreement with our explanation, Sun and Thomas (2013) proposed that participants engaging in a cooperative task with a friend experienced motor resonance when watching others’ actions. Thus, an alternative explanation for our data may be that the joint-action conditions of Experiment 1 led participants to incorporate the partner's hand into their own body representations and thus to prioritize the experimenter-pointed arrays.

A second alternative explanation comes from a series of studies showing that the covert activation of the motor programs corresponding to the partner’s actions requires a strong response inhibition in order to prevent participants from responding to the co-actor relevant stimuli (Sebanz, Knoblich, Prinz, & Wascher, 2006b; Tsai, Kuo, Jing, Hung, & Tzeng, 2006). Sebanz et al. (2006b), for example, found that the amplitude of the No-go P300 (a component reflecting action control and response inhibition: e.g., Bokura, Yamaguchi, & Kobayashi, 2001) was larger in the joint-action condition than in the individual condition. A later fMRI study by Sebanz, Rebbechi, Knoblich, Prinz, and Frith (2007) confirmed these findings by showing that the no-go trials, in which the co-actor was expected to respond, elicited greater activity in the inferior and superior parietal lobe as well as in the supplementary motor area (BA 6) in the co-action condition than in the individual condition. Sebanz et al. (2007) interpreted these results as an indication for increased demands on response inhibition during co-action. Based on these results, a different account for our data could be that the necessity to inhibit one’s own responses during no-go trials led participants to devote more processing time to the experimenter-relevant stimuli: under this account, the higher frequency and durations of fixations to the experimenter-pointed arrays would represent a byproduct of the selection conflict occurring in the joint-action condition (see Tsai et al., 2006, for discussion).

A similar hypothesis has been recently advanced by Elekes, Bródy, Halász, and Király (2016) to explain the so-called ‘joint memory effect.’ In the basic paradigm, introduced by Eskenazi, Doerrfeld, Logan, Knoblich, and Sebanz (2013), participants performed a categorization task either alone or together with a partner, followed by a surprise recall test. The words included in the encoding list belonged to three different categories, such that, in the joint-encoding condition, each participant in a pair pressed a prespecified key when he/she saw words of one category (e.g., one person responded to animals, the other to household items). Words of a third category did not require a response and served as control items. The key finding reported by Eskenazi et al. (2013) was that both the words responded to by the participants and those responded to by their co-actor were recalled better than control words. The authors proposed that this joint memory effect resulted from the motor simulation of the co-actor’s responses, which in turn led to the formation of more recallable memory traces. This hypothesis was later questioned by Elekes et al. (2016), who found enhanced recall performance to stimuli relevant to the co-actor also when the participants’ task required non-motor responses (counting the target words). To account for these findings, they speculated that, in the joint memory paradigm, it took longer for participants to decide whether they should respond to words from the other-relevant category (as it was associated with the co-actor’s task) than to words from the non-task-relevant category, and that the longer processing time resulted in a deeper encoding of the former category. Clearly, our data cannot be used to discriminate between these alternative explanations: additional studies are needed to determine whether, in the joint-action condition, the presentation of the experimenter-relevant arrays triggers the activation of brain areas involved in the inhibition of motor responses.

In the present study, we started from the assumption (supported by previous studies: Chum et al., 2007; Dodd & Shumborski, 2009) that performing pointing movements towards the to-be-remembered locations enhanced spatial memory, relative to a condition of passive observation. Moving from these findings, we asked ourselves whether the advantage could be extended to the recognition of the locations pointed by the experimenter in a joint-action condition. However, in doing so, we chose to set aside at least two central questions. The first question is whether the advantage is exclusively produced by pointing movements. That is, could different manipulations, such as counting the number of items or tapping one’s own foot, produce similar benefits to recognition memory? Evidence from different manipulations suggests that this could be the case. For example, in the Attentional Boost Effect (Spataro, Mulligan, & Rossi-Arnaud, 2013), participants press the spacebar whenever a red square appears on the screen and do nothing when the square is green: the key finding is that words or images paired with red squares are later remembered better than words or images paired with green squares. Intriguingly, the same advantage has been obtained when participants covertly counted the number of red squares appearing on the screen (Makovski, Jiang, & Swallow, 2013; Mulligan, Smith, & Spataro, 2016), suggesting that the need to modify an ongoing activity results in improved memory for co-occurring stimuli. However, although we cannot definitely rule out this hypothesis, unpublished evidence from our laboratory (Bhatia & Rossi-Arnaud, 2020; Bhatia, Spataro & Rossi-Arnaud, 2019a) suggests that the positive effects of pointing movements on spatial memory are not easily reproduced by different manipulations. For example, in one experiment using the original paradigm devised by Chum et al. (2007), a cartoon hand appeared below the locations of the move arrays and pointed towards each item (the no-move arrays were unchanged, with participants passively observing them); in another experiment, a series of black and white smileys appeared in the to-be-remembered locations of the move arrays, likely increasing their perceptual distinctiveness: nonetheless, in both cases we found that move arrays were recognized no better than no-move arrays. Lastly, in a final experiment, participants’ hands were passively moved towards the locations of the move arrays by the experimenter, mimicking self-performed pointing movements: yet, recognition memory was unaffected by passive movements (replicating the present conclusions, we showed that passive movements enhanced recognition memory only when they were randomly alternated with active pointing movements) (Bhatia, Spataro, & Rossi-Arnaud, manuscript in preparation). Taken together, these data suggest that the positive influence of pointing movements on spatial working memory is rather unique and is not due to factors such as perceptual distinctiveness or motor feedback. The second important question is that we did not include a control condition to determine whether performing pointing movements during the P-cued trials boosts spatial memory in the E-cued trials irrespective of the pointing movements performed by the experimenter. That is, what happens if participants point towards the locations of the move arrays in the P-cued trials but no one points during the E-cued trials: does a memory benefit occur even in this condition? The question is legitimate because in our experiments pointing movements were performed towards the same items (circles or squares) in all trials, and if circles (or squares) in the P-cued trials become distinctive because of the association with self-performed movements, then they might continue to be distinctive in the E-cued trials, even when the experimenter does not point to them. Future studies should test the validity of this hypothesis, for example by randomly varying the type of shapes to which participants must point.

To summarize, despite these limitations, the present study successfully replicated the results already reported by Bhatia et al. (2019b), showing that pointing movements were beneficial to recognition memory when the participants and the experimenter alternated in making pointing movements (Experiment 1), but not when the participants passively observed the movements performed by the experimenter (Experiment 2). Most crucially, we found that, in Experiment 1, both the number and the duration of fixations were higher for move than for no-move arrays, irrespective of the agent who performed the pointing movements; in contrast, no difference between the two types of arrays was obtained in Experiment 2. These data are consistent with the idea that one’s own and others’ actions were coded at the same representational level in the joint-action condition and were therefore associated with a similar pattern of eye fixations. Alternatively, the stimuli presented near the experimenter’s hand could be prioritized in the joint-action condition (Sun & Thomas, 2013) or the prolonged time necessary to inhibit the participants’ responses to other-relevant stimuli might incidentally improve their encoding (Elekes et al., 2016). In both cases, the outcome would be a better recognition of the experimenter-pointed arrays in the joint-action condition (as compared to no-move arrays).
